# Anonymized but Useful Synthetic Tabular Health Data for AI based Fall Risk Assessment

**DOI:** 10.1038/s41597-026-07910-z

**Published:** 2026-07-24

**Authors:** Ivana Nanevski, Sebastian Jäger, Maryam Mohebi, Matthias Schulte-Althoff, Jörg Pohle, Nicholas Chandler, Rahel Gubser, Alessia Nowak, Fabian Prasser, Daniel Fürstenau, Felix Balzer, Felix Biessmann

**Affiliations:** 1 Berliner Hochschule für Technik, Berlin, Germany; 2 Freie Universität Berlin, Berlin, Germany; 3 Charité - Universitätsmedizin Berlin, Berlin, Germany; 4 Alexander von Humboldt Institute for Internet and Society, Berlin, Germany; 5 Berlin Institute of Health at Charité - Universitätsmedizin Berlin, Berlin, Germany; 6 Einstein Center Digital Future, Berlin, Germany

## Abstract

Artificial Intelligence (AI) bears potential for improving health care, but this depends on the availability of open-access, realistic, and useful data. To facilitate AI model development in health care we release SynTabFall, a novel synthetic dataset for fall risk assessment. With a total of 745,380 samples and 44 attributes such as demographics, diseases, mobility and cognition related risk factors, this tabular dataset allows for training fall risk prediction models without access to the original patient data. Models trained on our synthetic dataset can reach predictive performance scores in fall risk assessment which are on par with models trained on real data. To support others in sharing data we also describe a process that was developed over multiple years in one of Germany’s largest hospitals in close collaboration between data protection officers, health care staff, informaticians and AI engineers. The proposed data sharing approach combines established methods for anonymization and modern generative AI (genAI) methods for synthesizing tabular data and allows for sharing health care data responsibly without sacrificing its utility. We release the synthetic fall risk dataset along with the software developed for synthetic data generation and evaluation.

## Background & Summary

With 28%–35% of individuals aged 65 years and older experiencing incidents of falling^1,2^, falls are the second leading cause of unintentional injury-related deaths globally. Novel AI models for better fall risk assessment can help to better support nursing care staff, relatives and patients to prevent falls. Such models can be improved in the research community through the availability of open-access, realistic and privacy-preserving health data. In this study we present SynTabFall^3^, a generated synthetic tabular dataset based on an anonymized German healthcare tabular dataset suitable for AI model development. The synthetic dataset counts 745,380 samples and 44 attributes. The task considered here is fall risk prediction and is described in more detail in^4^. The inspiration for this paper and data release originated in^5^ where the synthetic dataset was included in an end-to-end data generation and evaluation. The dataset consists mainly of attributes such as demographics, diseases, mobility and cognition related risk factors which are already used in practice. Releasing such data, even in synthetic form, requires strict compliance with privacy and data protection regulations, which poses considerable challenges. The legal debate on personal data, anonymization, and anonymized data under the EU General Data Protection Regulation (GDPR)^6^ views anonymization as one or more spectra. These range from “de-identification,” “low-level anonymization,” or “relative anonymization”, which may be sufficient as data minimization or security measures, to “complete,” “perfect,” or “absolute” anonymization^7–11^. When it comes to achieving perfect anonymization, the GDPR requires implementation “in an effective manner” (Art. 25^6^). As practical examples how this can be implemented are rare, balancing patient privacy with the utility of anonymized training data remains a persistent challenge. To address this, we describe a data sharing process developed over several years at one of Germany’s largest hospitals. This process benefited from close collaboration among informaticians and AI engineers, healthcare professionals as well as data protection officers.

The proposed data sharing approach combines established methods for anonymization and modern generative AI (genAI) methods for synthesizing tabular data and allows for sharing health care data responsibly without sacrificing its utility. In a multi-step procedure patient data is first anonymized, then the number of variables is further reduced during AI model development to focus on relevant variables only. In a last step, a suite of tabular genAI methods are evaluated with respect to fidelity, utility and privacy to select the best genAI methods at the Pareto-front of the multi-objective optimization trading off patient privacy and utility for AI model development.

To the best of our knowledge, the dataset we are releasing is one of the first large-scale, anonymized synthetic German healthcare tabular dataset suitable for AI model development to be made publicly available. We believe that we can support further research improving the state-of-the-art in the application scenario considered here, fall risk assessment, but we hope that the processes will enable researchers to release similar datasets beyond this application scenario. To help other researchers responsibly share health data, we provide the software we developed which documents all necessary details to replicate our approach in other hospitals or non-health contexts.

## Methods

**Fig. 1:**
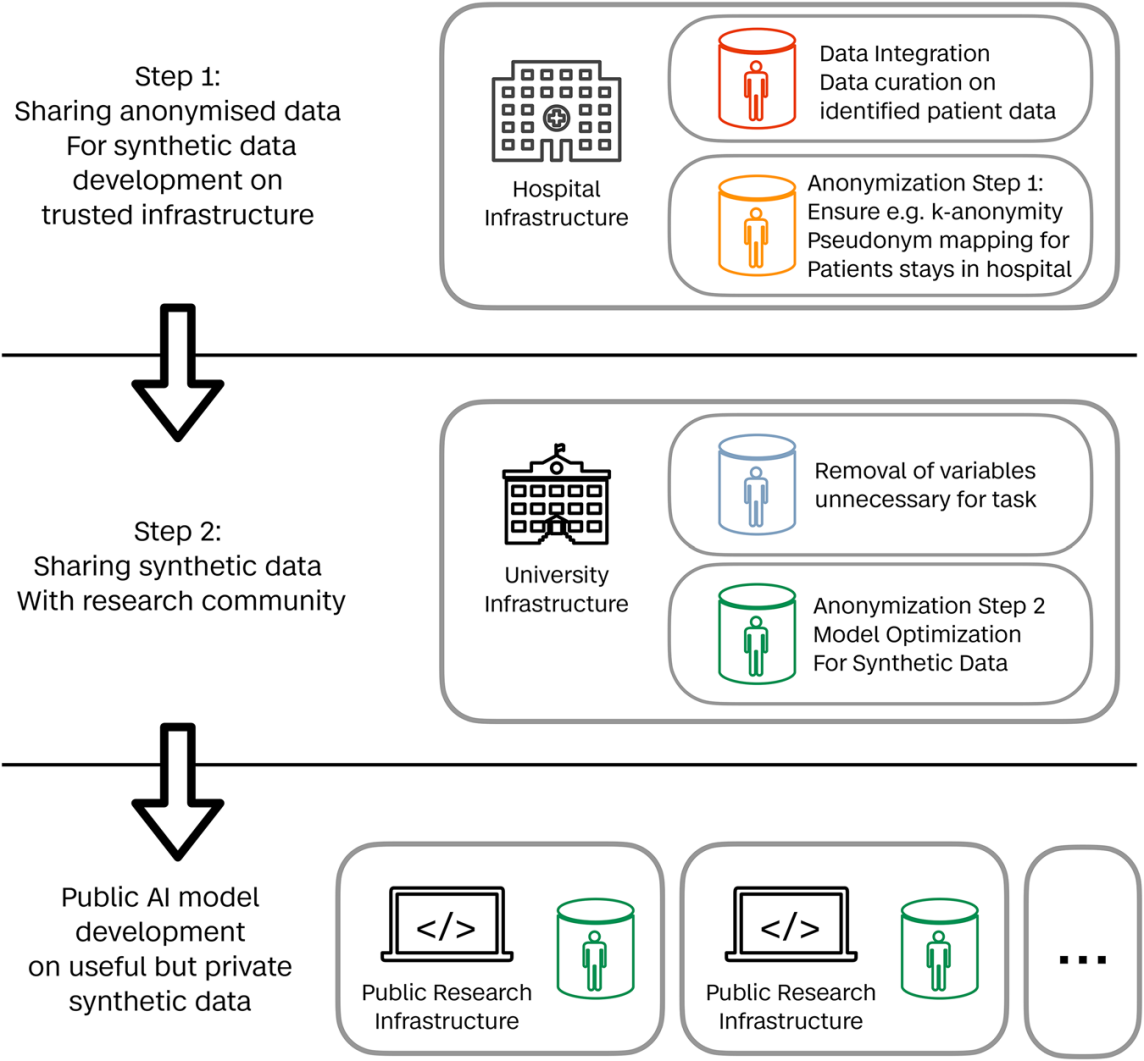
Schematic illustration of the proposed data sharing process.

In the following section, we describe the data preparation process which can be structured in three steps as illustrated in Fig. 1. In a first step, detailed in the Initial Data Anonymization section, the original data is curated, only essential variables were selected and if necessary transformed to mitigate privacy attack risks. Patient IDs were pseudonymized prior to the data export from the hospital. Note that the re-identification mapping to revert pseudonyms to real patient IDs stayed within the hospital and was never shared with the researchers conducting the synthetic data generation. Hence for the researchers the training data is already anonymized when leaving the hospital according to recent EU legislation^12^. In a second step, described in the Synthetic Data Development section, the data is transferred to a university or research institute that has secured infrastructure for further anonymization and model development for training genAI models to ultimately synthesize the data that will be shared. Finally, in a third step the synthetic data is shared with the research community to foster faster AI innovations in the medical field. For the purpose of this retrospective study, informed consent was waived by the ethics board. The consent waiver did not permit the sharing of personally identifiable information; instead, the study generated synthetic data derived from highly anonymized data, which do not qualify as personally identifiable information. For more information we refer to the Ethics statement and Data availability sections.

### Original data description

The attributes contain standard demographic variables, such as age and gender, medical information, such as existing diseases or therapeutic measures, attributes summarizing the cognitive and physiological condition of patients, and medical items that are relevant for nursing care, such as the mobility of patients, and fall related information including risk factors. The label, fallen, is a binary variable indicating whether a patient has experienced a fall. To evaluate the patients’ risk of falling, the hospital adheres to the patient classification system introduced by Jones^13^, which assesses the patients’ independence in daily life activities, such as feeding, personal toilet, and walking. A complete list of data attributes and their respective types are listed in Supplementary Table 1. In Table 1 we present the prevalence between patients who have or have not experienced fall in the original dataset, both overall and demographic groups. These statistics were computed from the original dataset with 931,726 samples. The dataset was further split into train (745,380 samples) and test (186,346 samples) sets. The synthetic dataset presented in this study was generated from the train set, while the 186,346 samples in the test set were reserved exclusively for evaluation. We will further refer to these splits as original train set and original test set.

**Table 1:** Original dataset: prevalence of fallers and non-fallers in the original dataset, across all patients and within demographic groups.

Characteristics		All patients, n (%)	Fallers, n (%)	Non-fallers, n (%)
All samples		931,726 (100)	10,442 (1.12)	921,284 (98.88)
Age Range	10–20	3,349 (100)	3 (0.09)	3,346 (99.91)
	20–30	83,926 (100)	144 (0.17)	83,782 (99.83)
	30–40	155,611 (100)	316 (0.20)	155,295 (99.80)
	40–50	142,956 (100)	427 (0.30)	142,529 (99.70)
	50–60	144,232 (100)	846 (0.59)	143,386 (99.41)
	60–70	135,627 (100)	1,577 (1.16)	134,050 (98.84)
	70–80	118,549 (100)	2,366 (2.00)	116,183 (98.00)
	80–90	100,742 (100)	3,061 (3.04)	97,681 (96.96)
	90–100	40,067 (100)	1,449 (3.62)	38,618 (96.38)
	100–110	6,445 (100)	249 (3.86)	6,196 (96.14)
	110–120	222 (100)	4 (1.80)	218 (98.20)
Sex	female	496,560 (100)	4,696 (0.95)	491,864 (99.05)
	male	435,166 (100)	5,746 (1.32)	429,420 (98.68)

Statistics are calculated using the full original dataset before the train-test split.

### Data preparation

For preparing the data all relevant tables from the hospital information system (admissions, demographic data, nursing assessments, fall incident documentation, diagnoses, procedures and medication records) were linked at the level of inpatient stays using pseudonymous patient identifiers. The dataset was then restricted to completed inpatient stays within the observation period that contained at least one fall risk assessment and complete basic demographics (sex, age, admission and discharge timestamps). Records with inconsistent or implausible information, such as duplicated encounters, were removed. Second, diagnosis, procedure, and medication codes were aggregated into clinically meaningful groups. Finally, we performed descriptive data quality checks (completeness, plausibility, distributional comparisons across wards and calendar years) in collaboration with clinical experts and restricted the working dataset to variables that were either necessary for cohort definition and outcome construction or judged to be clinically relevant predictors of in-hospital falls.

### Initial data anonymization

Developing synthetic datasets requires iterative model development of generative AI components. The infrastructure for these tasks is not always available in health care institutions, in particular smaller hospitals or nursing care facilities. To enable easier model development and faster development cycles, we first anonymized the data in the secure processing environment of the hospital. Building on the prepared dataset described in the Data Preparation section, we conducted anonymization steps before transferring the data to an external research environment to reduce the risk of exposing sensitive personal information. In particular, direct identifiers such as names, exact addresses, insurance numbers and internal patient identifiers were removed from the analytical dataset. Dates of birth and exact calendar dates were replaced by less granular representations (for example, age in 10-year bands or relative timing within the hospital stay), and location information was generalized to broader organizational units. Free-text fields and other unstructured content with a high likelihood of containing identifying information were excluded. Categorical variables with a long tail of very infrequent levels, such as rare diagnoses, medical procedures, and medications, categories were aggregated into higher-level clinical groupings.

### Removal of unnecessary variables

A central element of responsible data sharing is to restrict the number of attributes to a minimum. This ensures to mitigate unnecessary risks of sensitive data leakage, while ensuring high utility. The drawback is that this approach requires a measure of utility or in other words a task associated with the data. In this work, we assume that the associated task is fall risk assessment. For more details on the task we refer to^4^. We applied several approaches to reduce the number of attributes in the dataset, which can be broadly categorized into two groups. For one, after the initial data anonymization (Section Initial Data Anonymization), clinical staff manually filtered out variables that were not related to the task considered, fall risk prediction. An alternative approach was based on automatic feature selection using eXplainable AI (XAI) methods. Concretely, we trained a classification model to predict patients’ fall risk on all variables that were not manually filtered out by clinical staff. We then tested the feature importance of individual variables for fall risk assessment. However, since the resulting selection differed from variables commonly used in fall risk assessment tests, we did not omit any additional variables. As we believe this task specific data driven variable selection process is still useful we included it here nonetheless. For more details we refer to^4^. In Fig. 1, we highlighted the variable selection in the box of the university researchers, in practice the above described procedures are often distributed across clinical staff, who have more complete and faster data access and know the task better, and university staff, who can use XAI based filtering procedures more efficiently.

### Synthetic data development

For data generation we optimized over 9 models, representing the most common generative model architectures for tabular data. These include Gaussian and Copula based models, GAN-based, Adversarial Random Forest (ARF), diffusion models and differentially private (DP) models. For most models, we use the implementations provided by the Synthetic Data Vault (SDV) package^14^, which are encapsulated in Synthius^15^. For TabDiff, we follow the implementation of^16^ and for PATEGAN, the implementation of^17^. As a result, we obtained 9 different synthetic datasets, each generated by a different generative model and evaluated across fidelity, utility, and privacy (Supplementary Table 2).

To reduce computational load during the model selection process, we subsampled 50,000 samples of the data, while still keeping the original target distribution. However, after we chose the Pareto-optimal generative model, we trained the best generative model again on the original dataset. We split the dataset into two sets, train (745,380 samples) and test (186,346 samples) sets. The generated synthetic dataset therefore contains 745,380 samples.

### Ethics statement

The ethics committee of the Charité - Universitätsmedizin Berlin approved the analysis of Charité data (EA2/184/21, Amendment vote Dec 2, 2024). From this analysis, the original dataset was derived which was further used for generating the synthetic dataset that we release with this paper. Restricted conditions apply. The original data were anonymized through the institution’s Health Data Platform and subsequent further anonymization steps (e.g., removing irrelevant fields). The data protection officers and the clinical trial office of the Charité (CTO-22-0838) and federal data protection officers were consulted for their advice.

## Data Record

The dataset is publicly available on Zenodo under a CC BY 4.0 license^3^. The data schema includes general demographics such as sex and age and a total of 44 attributes that can be broadly categorized into disease related information, fall related attributes, including the target column - fallen, nursing care related attributes, medical items and cognitive attributes (Supplementary Table 1). The primary purpose of the dataset is to preserve the original downstream application of the original source data, which is fall risk assessment based on the provided clinical attributes.

### Disease related attributes

These attributes cover prior diseases, diagnoses and procedures described with the standardized International Statistical Classification of Diseases, Tenth Revision, German Modification (ICD-10-GM)^18^ and the OPS procedure classification (derived from the International Classification of Procedures in Medicine; ICPM)^19^ systems accordingly.

### Fall related attributes

The label column is a binary feature (*fallen*) which informs if the patient has fallen in the hospital. Accordingly, a *fall-risk* is assigned mostly by nursing care personnel or medical professionals. These values can be 0 (no risk), + (increased risk), or ++ (high risk). In line with the fall-risk information the dataset provides information if the patient had fallen in the last 12 months (*fall-last_12_month*) and if so, how often (*fall-how_many_last_12_month*), if they have fallen while being admitted in the hospital (*fall-while_stay*) or while being transferred (*fall-while-transfer*).

### Nursing care related attributes

These contain information relevant for patient care. There are indicators if a patient had decubitus at the moment of assessment (*decubitus-at_the_moment*), decubitus on admission (*decubitus-admission*) and potential risk for decubitus (*decubitus-risk*) with possible values of 0 (no risk), + (increased risk), or ++ (high risk). Furthermore, bed mobility related information is provided, such as whether the patient is impaired in his or her bed mobility (*bed_mobility-impairment*), and if so to which degree measured by the Jones Score (*bed_mobility-jones*), and whether this immobility is connected to his or her skin condition (*bed_mobility-skin_condition*). All Jones related columns (which assesses the patients’ independence in daily life activities) have the following values: 0 (independent), 1 (largely independent), 2 (partially independent), 3 (minimally independent), 4 (A or B) (not independent/dependent). For level 4 particularly, the medical professionals differentiate between 4 A where a patient is defensive for getting help and 4B which means that a patient is completely motionless. Each record also contains information on whether the patient is impaired for transfers within the hospital (*transfer-impairment*), and if so, to which degree measured by the Jones Score (*transfer*). More attributes relevant for patient care cover excretion related information such as general excretion impairments (*excretions-impairment)*, or incontinence (*excretions-incontinence*) or nocturia (*excretions-nykturie*)

### Medical item attributes

These attributes cover different medical items that a patient might be wearing for improved living conditions such as arm or leg splint (*medical_items-arm_or_leg_splint*), atrioventricular systems (*medical_items-A_V_system*), plaster or neck brace (*medical_items-plaster_or_neck_brace*), compression socks for improved blood circulation (*medical_items-compression_stocking*), oxygen/ventilation mask for improved respiratory function (*medical_items-ventilation_mask*), glasses (*medical_items-O2_glasses*), a prosthetic implant (*medical_items-prosthesis*), orthosis (*medical_items-orthesis*), and if they got some skin condition while a medical item was applied on them (*medical_items-skin_condition_at_item_application*). Then there are the walking related attributes, such as whether or not there is an impairment (*walk-impairment*), and if so, the degree of the impairment measured by the Jones Score (*walk-jones*), whether they have impaired balance or gait while walking (*walk-balance_and_gait_impaired*), and whether they use a walking aid (*walking_aid*).

### Cognitive attributes

Attributes related to cognitive abilities include information on potential impairment (*cognition-impairment*). More specifically, whether the patient is agitated (*cognition-agitated*), confused (*cognition-confused*), whether the impairment is related to the patient’s relation to time (*cognition-disoriented_time*), location (*cognition-disoriented_location*), or his or her own self (*cognition-disoriented_own_person*). The dataset further includes information about whether a patient intakes psychotropic or sedatives drugs (*psychotropic_or_sedatives_drugs*).

## Data Overview

The synthetic dataset was generated from the original train set of the original data (745,380 samples), as explained in Section Original Data Description. The generated synthetic dataset has 745,380 samples, 7,276 (0.98%) of which are labeled as fallers (Table 2), which is relatively close to the original data (1.12%). The dataset itself is heavily imbalanced with respect to the target attribute. However, since the synthetic data preserves the original class distribution, achieves comparable macro-level utility metrics, and demonstrates relatively high fidelity scores, we believe that the underlying data structures and relationships are largely preserved. For more details we refer to Supplementary Table 2.

**Table 2:** Released synthetic dataset (generated from the original train set): prevalence of fallers and non-fallers in the synthetic dataset, across all patients and within demographic groups.

Characteristics		All patients, n (%)	Fallers, n (%)	Non-fallers, n (%)
All samples		745,380 (100)	7,276 (0.98)	738,104 (99.02)
Age Range	10–20	2,644 (100)	2 (0.08)	2,642 (99.92)
	20–30	81,011 (100)	133 (0.16)	80,878 (99.84)
	30–40	122,544 (100)	292 (0.24)	122,252 (99.76)
	40–50	93,222 (100)	396 (0.42)	92,826 (99.58)
	50–60	112,176 (100)	702 (0.63)	111,474 (99.37)
	60–70	112,913 (100)	1,054 (0.93)	111,859 (99.07)
	70–80	96,133 (100)	1,509 (1.57)	94,624 (98.43)
	80–90	85,742 (100)	1,945 (2.27)	83,797 (97.73)
	90–100	33,533 (100)	1,030 (3.07)	32,503 (96.93)
	100–110	5,081 (100)	194 (3.82)	4,887 (96.18)
	110–120	381 (100)	19 (4.99)	362 (95.01)
Sex	female	398,611 (100)	3,344 (0.84)	395,267 (99.16)
	male	346,769 (100)	3,932 (1.13)	342,837 (98.87)

**Fig. 2:**
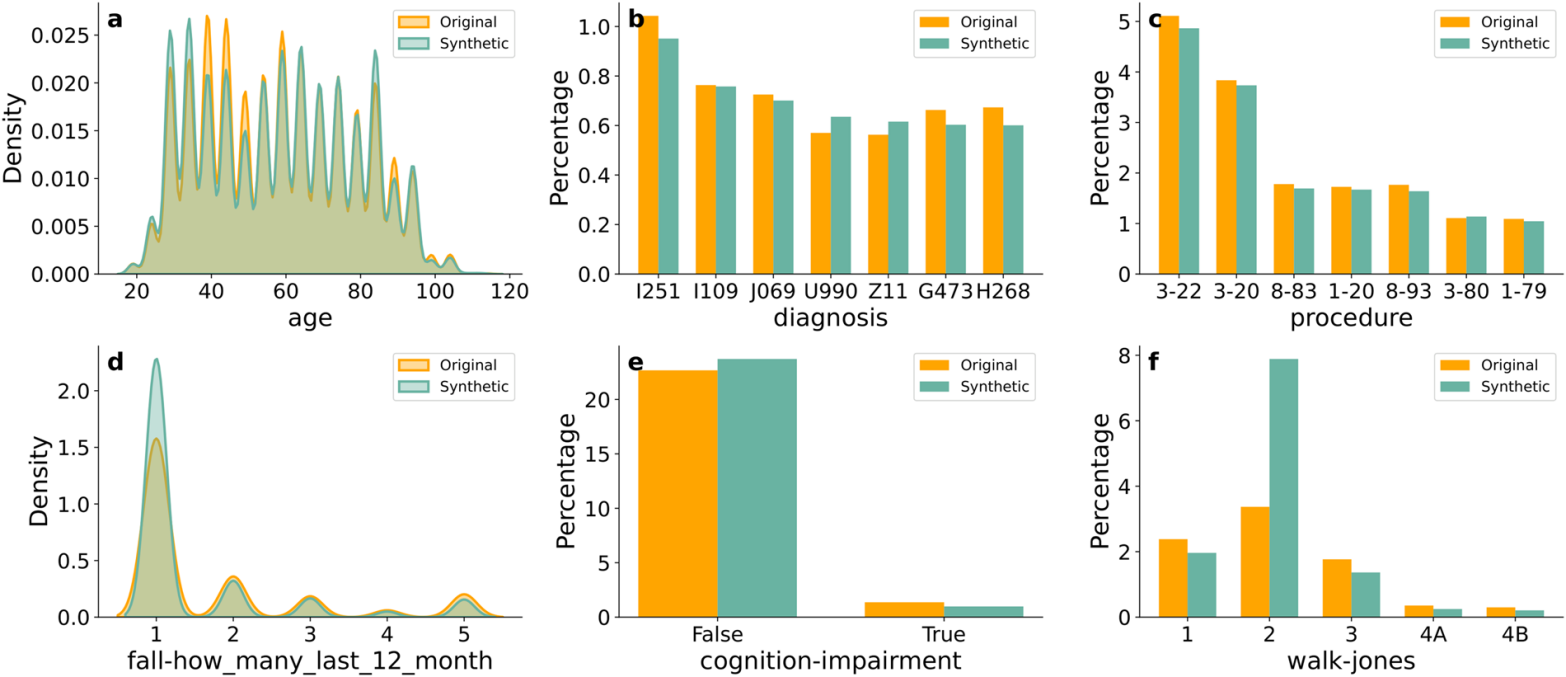
Marginal distribution comparisons between the original and synthetic dataset on a subset of variables. Missing value statistics for columns with high ratios of missing data were omitted for better readability.

The synthetic dataset counts 398,611 (53.5%) females and 346,769 (46.5%) males, whereas in the original dataset these are 53.3% for females and 46.7% for males. The age-wise marginal distributions are also comparable between the original and synthetic datasets (Fig. 2(a)).

Of all variables, 23 (52%) contain missing values. The feature medical_items-skin_condition_at_item_application (96.65%) holds the highest missingness proportion, while diagnosis has the lowest proportion of missing values (12.32%). Overall, the synthetic dataset preserves the missingness distribution of the original dataset. For the overall dataset schema and details on the missing data, we refer Supplementary Table 1.

## Technical Validation

In order to select the optimal generative model for the synthetic dataset, we evaluated three key metrics: fidelity, utility and privacy^15^. The evaluation protocol is described in detail in^5^. For the evaluation we included 33 metrics, 13 of which represent fidelity, 19 describe privacy, and one - F1 macro is our utility metric. We perform the analysis using well-maintained libraries, including SDMetrics^20^, extended where necessary with open-source tools such as Autogluon^21^, scikit-learn^22^, XGBoost^23^, and Anonymeter^24^.

### Fidelity

Fidelity quantifies how closely the synthetic data resemble the real data^25,26^. In practice, fidelity is measured on multiple levels: (a) univariate fidelity, which compares the similarity of single attributes, (b) bivariate fidelity, which compares second-order statistics, such as correlations of pairs of attributes, and (c) multivariate fidelity which evaluates the resemblance of the joint distribution across all attributes. Furthermore, to better capture the multivariate similarity, we used the propensity score, which evaluates how well a ML model can distinguish the synthetic data from the original data on hold-out test data. Finally, we evaluated whether the generative model generates new, unique records (novelty test)^27,28^.

### Utility

Utility measures whether the synthetic data can support downstream machine-learning tasks. A standard approach includes training two models, one using the original training data and another using the synthetic data, with both models evaluated on a hold-out test dataset from the original data (Training on Real, Testing on Real (TRTR) and Training on Synthetic, Testing on Real (TSTR)). When the two models achieve a comparable predictive performance, the synthetic data can be considered as a high utility dataset, a reliable proxy for the real data on downstream tasks^25,29–32^. For that purpose, we used AutoGluon^21^, a well maintained AutoML library that integrates most ML model classes and supports an end-to-end hyperparameter optimization. Comparing predictive performance could be quantified with various metrics, like accuracy, precision, recall, F1-score. In this study, we used the F1 macro score for assessing utility. Additionally, as a baseline reference for future model development on this synthetic dataset we refer to the results obtained from training an AI model on the original train set and tested on the original test set. We report an f1-score, macro avg of 0.61 on the test split (Table 3, Data = Original).

**Table 3:** Classification performance on the original test set of an AI fall risk model trained on the original train set (Original) and a model trained on the synthetic data (Synthetic).

Data		Fall	No Fall	Macro Avg
Original	f1-score	0.23	0.99	0.61
	precision	0.39	0.99	0.69
	recall	0.16	1.00	0.58
	support	2,088	184,258	186,346
Synthetic	f1-score	0.13	0.99	0.56
	precision	0.13	0.99	0.56
	recall	0.12	0.99	0.56
	support	2,088	184,258	186,346

Note that the recall metric is most relevant for fall risk assessment, and additionally, macro-averaged metrics are more informative regarding the imbalances target class. These metrics are similar between both models, indicating high utility of the synthetic data.

### Privacy

Privacy is the most critical metric in healthcare data^33^, and can be assessed with analyses that simulate different attack scenarios. These include a) re-identification attacks, where synthetic records are matched to real individuals, b) attribute inference attacks, where an adversary attempts to infer unknown attributes of a target from the synthetic data, c) membership inference attacks^34^, where adversaries try to determine whether an individual belongs to the training data, and d) data-linkage attacks, where synthetic data are used to link attributes between two disjoint datasets derived from the original data. In addition, distance-based metrics are used to compare synthetic and original records. These include distance to closest record (DCR), and nearest neighbor distance ratio (NNDR)^35^. To evaluate privacy, we used four types of privacy metrics: distance-based metrics (from SDMetrics^20^), attribute inference attack (from SDMetrics and anonymeter), and singling out and linkability, both from anonymeter^24^. In addition we also worked with the data protection authorities of the state of Berlin to develop novel protocols that would ensure that the risks of re-identification attacks are fair across subgroups of patients.

### Evaluation results and model selection

In Supplementary Table 2, we show the utility, fidelity and privacy metrics. The evaluation protocol is detailed in^5^. TabDiff shows to be the best choice of model that balances between utility/fidelity and privacy. Based on these results we decided to release the synthetic dataset generated with TabDiff^16^, a mixed-type diffusion framework for tabular data generation which handles the high heterogeneity across different feature distributions with feature-wise learnable diffusion processes. To compare the newly generated dataset of 745,380 samples, we provide the classification report from a model trained on the synthetic data, and tested on the original test data split, and report an f1-score, Macro Avg of 0.56 in Table 3 (Data = Synthetic), in comparison to the performance of the original dataset, a f1-score, Macro Avg of 0.61 (Table 3) (Data = Original).

## Supplementary information

Supplementary Table 1

Supplementary Table 2

## Data Availability

The generated synthetic datasets is publicly available on Zenodo under a CC BY 4.0 license (https://zenodo.org/doi/10.5281/zenodo.20427694). The dataset includes synthetic tabular hospital data with 44 attributes covering patient demographics (age and sex), diagnoses and procedure, nursing care attributes, medical items, and cognition related attributes. The task bound to this dataset is fall risk prediction, therefore the dataset includes a binary fall outcome label - fallen, and fall history. Diagnoses and procedure information is encoded using standardized classification systems (ICD-10- GM and OPS). The dataset is fully synthetic and contains no real patient records. This dataset is published for the first time as part of this study. The original dataset was provided by Charité – Universitätsmedizin Berlin and contains sensitive patient-level data. Therefore, the dataset is not publicly available. Researchers interested in accessing the data for the purpose of reproducing the analyses may contact the corresponding author at daniel.fuerstenau@charite.de. Requests will be considered on a case-by-case basis and are subject to approval by Charité, compliance with applicable data protection regulations, and, where required, the execution of appropriate data use or data transfer agreements.

## Code availability

The software used for the data generation and evaluation is available on GitHub, under https://github.com/calgo-lab/Synthius.
